# Epidemiological survey and genetic diversity of *Bartonella* in fleas collected from rodents in Fujian Province, Southeast China

**DOI:** 10.1186/s13071-024-06305-6

**Published:** 2024-06-18

**Authors:** Shuheng Zhou, Yuwei Nian, Zhiwei Zeng, Tengwei Han, Weijun Liu, Kuicheng Zheng, Fangzhen Xiao

**Affiliations:** 1 Fujian Provincial Key Laboratory of Zoonosis Research, Fujian Center for Disease Control and Prevention, Fuzhou, Fujian China; 2 The School of Public Health, Fujian Medical University, Fuzhou, Fujian China

**Keywords:** *Bartonella*, Fleas, Prevalence, Gene diversity, PCR

## Abstract

**Background:**

Fleas, considered to be the main transmission vectors of *Bartonella*, are highly prevalent and show great diversity. To date, no investigations have focused on *Bartonella* vectors in Southeast China. The aim of this study was to investigate the epidemiological and molecular characteristics of *Bartonella* in fleas in Southeast China.

**Methods:**

From 2016 to 2022, flea samples (*n* = 1119) were collected from 863 rodent individuals in seven inland and coastal cities in Southeast China. Flea species, region, gender, host species and habitat were recorded. The DNA samples from each individual flea were screened by real-time PCR for the *Bartonella ssrA* gene. All positive samples were confirmed by PCR based on the presence of the *gltA* gene and sequenced. The factors associated with *Bartonella* infection were analyzed by the Chi-square test and Fisher's exact test. ANOVA and the t-test were used to compare *Bartonella* DNA load.

**Results:**

*Bartonella* DNA was detected in 26.2% (293/1119) of the flea samples, including in 27.1% (284/1047) of *Xenopsylla cheopis* samples, 13.2% (5/38) of *Monopsyllus anisus* samples, 8.3% (2/24) of *Leptopsylla segnis* samples and 20.0% (2/10) of other fleas (*Nosopsyllus nicanus*, *Ctenocephalides felis*, *Stivalius klossi bispiniformis* and *Neopsylla dispar fukienensis*). There was a significant difference in the prevalence of *Bartonella* among flea species, sex, hosts, regions and habitats. Five species of* Bartonella* fleas were identified based on sequencing and phylogenetic analyses targeting the *gltA* gene: *B. tribocorum*, *B. queenslandensis*, *B. elizabethae*, *B. rochalimae* and *B. coopersplainsensis*.

**Conclusions:**

There is a high prevalence and diversity of *Bartonella* infection in the seven species of fleas collected in Southeast China. The detection of zoonotic *Bartonella* species in this study, including *B. tribocorum, B. elizabethae* and *B. rochalimae,* raises public health concerns.

**Graphical Abstract:**

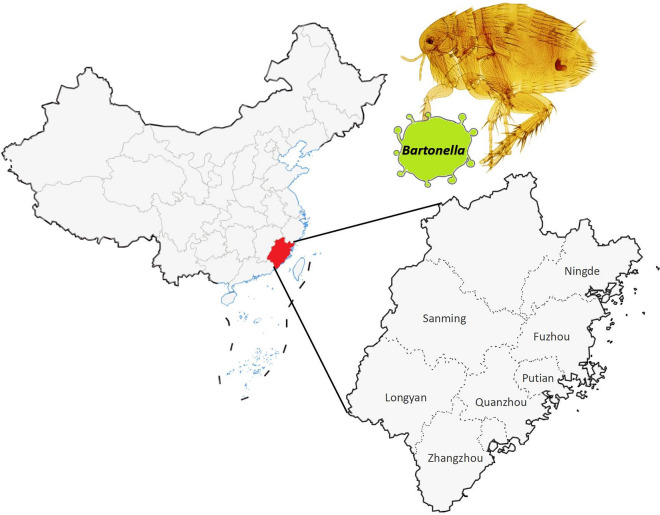

**Supplementary Information:**

The online version contains supplementary material available at 10.1186/s13071-024-06305-6.

## Background

*Bartonella* is a group of Gram-negative, fastidious, facultative, intracellular parasitic aerobic bacilli belonging to the class *Proteobacteria*, order *Rhizobacteria*, family *Bartonellaceae* and genus *Bartonella* that parasitize the erythrocytes and vascular endothelial cells of hosts and infect humans or other mammalian hosts through blood-sucking arthropods [1]. At least 40 species of *Bartonella* and its subspecies are currently recognized, of which at least 15 are human pathogens [2]. The clinical manifestations of *Bartonella* infection in humans range from mild to life-threatening and can be acute or chronic. Known symptoms of *Bartonella* in humans include endocarditis, myocarditis, fever and neurological disorders, intraocular retinitis, meningitis, splenomegaly and lymph node enlargement [3–7]. This constellation of nonspecific and variable symptoms make *Bartonella* infection difficult to diagnose clinically (Additional file 1: Table S1).

Rodents are natural hosts for approximately 20 species of *Bartonella* [8], and *Bartonella* has been detected in almost 100 rodent species worldwide. Importantly, a number of human pathogenic *Bartonella* species, such as *B. elizabethae*, *B. grahamii*, *B. vinsonii subsp. arupensis* and *B. washoensis*, are carried by rodents [9].

*Bartonella* is mainly transmitted horizontally [10], with arthropods acquiring *Bartonella* when blood feeding on an infected host with the subsequent transfer *Bartonella* to another host. Sand flies, body lice and cat fleas are involved in the transmission of *B. bacilliformis*, *B. quintana* and *B. henselae*, respectively [1]. Fleas are considered to be the primary vectors of *Bartonella* transmission among rodents, and a variety of fleas have been shown to be infected by zoonotic *Bartonella* species such as *B. henselae*, *B. clarridgeiae*, *B. quintana*, *B. grahamii* and *B. elizabethae* [11–15]. Fleas have been shown to play an important role in the transmission and acquisition of *Bartonella* species in rodents, and *Bartonella* DNA has been detected in fleas on rodents [16], providing evidence that fleas are vectors for the transmission of *Bartonella* among rodents.

Currently, 28 species of rodents belonging to seven families and 14 genera and 27 species of fleas belonging to six families and 18 genera have been identified in Fujian Province (China) [17, 18]. Previous systematic investigations conducted on 10 species of *Bartonella* host rodents harboring *Bartonella* in Southeast China identified *Bartonella* species in rodents, including *B. tribocorum*, *B. grahamii*, *B. rattimassiliensis*, *B. queenslandensis*, *B. elizabethae*, *B. phoceensis*, *B. coopersplainsensis*, *B. japonica* and *B. rochalimae* [19]. To date, however, no investigations have been conducted on *Bartonella* vectors. In the present study, we analyzed the epidemiological and molecular characteristics of *Bartonella* in fleas in Southeast China by investigating *Bartonella* infection in several areas of this region. Our aim was to assess the public health risk of the host-vector relationship between rodents and fleas on the transmission of *Bartonella* in the natural habitats of Southeast China.

## Methods

### Ethical aspects

This study was approved by the Ethics Committee of Fujian Center for Disease Control and Prevention (No: FJCDCNT1811-2015). All rodents were treated in accordance with the Guidelines of Regulations for the Administration of Laboratory Animals of the People's Republic of China.

### Sample collection and identification

Rodents were captured in seven inland and coastal cities in Southeast China, namely Zhangzhou City, Quanzhou City, Sanming City, Longyan City, Ningde City, Fuzhou City and Putian City, and one to three fleas were collected from the body surface of each captured animal. Rodents were captured in live-capture traps baited with corn. Live traps were placed every night at each surveillance point for three consecutive nights at locations where rodent activities were detected, and retrieved the following morning.

Following capture, rodents were anesthetized with ether, and fleas were collected from the body surface of the rodents and from the cloth bags in which the rodents were held. Chinese monographs were used to identify the species of trapped rodents according to body shape, tail, coat color and other morphological characteristics [20]. The fleas were identified to species under the stereomicroscope by observing the distribution of setae and spines and the morphology of important structures such as eyes and genitalia by stereomicroscope, as well as by literature references [21]. We then individually recorded flea species, region, sex, host species and habitat. The fleas were morphologically classified and counted for registration and were stored in 75% alcohol at -20 °C until examination. After fleas had been collected, all rodents were used for surveillance in other programs.

### Molecular analyses

Following published guidelines [22], before DNA extraction, each individual flea was immersed in 75% ethanol for 5–10 min, followed by two to three immersions in phosphate-buffered saline (PBS). The flea samples were then immersed in the lysate for 2 h and ground to a powder. DNA was extracted using a bacterial genomic DNA extraction kit (Tianlong Science & Technology, Xi'an, China) according to the manufacturer's instructions and stored at − 20 °C. DNA was extracted in order to identify *Bartonella* species using a real-time PCR (qPCR) assay targeting a transfer-mRNA gene (*ssrA*) [23]. The primers *ssrA*-F (5′-GCTATGGTAATAAATGGACAATGAAATAA-3′) and *ssrA*-R (5′-GCTTCTGTTGCTAGGTG-3′) and the FAM-labeled probe (FAM-ACCCCGCTTAAA CCTGCG-BHQ1) were used to amplify a 301-bp fragment of the *ssrA* gene. qPCR amplification was performed in a 20-μl reaction mixture containing 10 μl of Premix Ex Taq (Probe qPCR; Takara, Shiga, Japan), 0.4 μl each of 10 μM forward and reverse primers, 0.2 μl of 10 μM probe, 3 μl of DNA template and double-distilled water. The qPCR conditions were: 95 °C for 5 min; then 50 cycles of 95 °C for 15 s and 60 °C for 45 s. Samples with Ct (cycle threshold) values ≤ 35 were considered to be positive for *Bartonella* DNA. Positive samples were then subjected to conventional PCR to amplify the 379-bp *gltA* gene fragment [24] using the primers BhCS781.p (5′-GGGGACCAGCTCATGGT GG-3′) and BhCS1137.n (5′-AATGCAAAAAGAACAATAAACA-3′)[24]. The conventional PCR analysis was carried out in a total reaction volume of 25 μl containing 3 μl of template DNA, 1 μl each of 10 μM forward and reverse primers, 12.5 µl Premix Taq™ (Premix Taq Version 2.0 plus dye; Takara) and 7.5 µl double-distilled water. The amplification procedure was: 95 °C for 5 min; followed by 35 cycles of 95 °C for 30 s, 58 °C for 30 s and 72 °C for 30 s; with a final cycle at 72 °C for 5 min. The PCR products were separated by electrophoresis in a 1.5% agarose gel. During all PCR amplifications, distilled water was used as the negative control and positive DNA samples obtained from previous rodent surveys [19] were used as positive controls.

*Bartonella ssrA* sequences were sent to Sangon Biotech Company (Sangon Biotech, Shanghai, China) for gene synthesis to construct plasmid DNA. In addition, the *Bartonella* DNA load was calculated for each positive flea sample using a standard curve generated from a tenfold dilution (2log_10_-6log_10_ copies/μl) of plasmid DNA encoding a 300-bp *B. henselae ssrA* gene fragment.

### DNA sequencing and phylogenetic analysis

Positive amplification products were subsequently sent to Sangon Biotech Company (Sangon Biotech) for sequencing.

The *gltA* sequences were compared with the sequences of the type strains of the validated *Bartonella* species in the GenBank database using NCBI BLAST (https://blast.ncbi.nlm.nih.gov/Blast.cgi). After alignment of the *gltA* sequences by ClustalW, phylogenetic trees were created using the neighbor-joining method in MEGA 11.0 software. The best-fit nucleotide substitution model for the phylogenetic analysis was estimated based on the Bayesian information criterion (BIC) calculated using MEGA 11 software [25].

### Statistical analysis

The Chi-square test (*χ*^2^) and Fisher's exact test were used to evaluate the correlations between flea species, region, gender, host species, habitat and *Bartonella* infection. *P* < 0.05 was considered to indicate statistical significance. Analysis of variance (ANOVA) and the t-test were used to compare *Bartonella* loads.

All statistical analyses were performed using SPSS version 23.0 statistical software (SPSS IBM Corp, Armonk, NY, USA).

## Results

### Flea collection and morphological identification

A total of 1119 fleas were collected in seven cities during this survey, and seven species of fleas were identified (Table 1): *Xenopsylla cheopis* (*n* = 1047), *Monopsyllus anisus* (*n* = 38), *Leptopsylla segnis* (*n* = 24 ), *Ctenocephalides felis* (*n* = 6 ),* Nosopsyllus nicanus* (*n* = 1 ), *Neopsylla dispar fukienensis* (*n* = 1) and *Stivalius klossi bispiniformis* (*n* = 2). Among these, *X. cheopis* was the dominant flea species collected from the rats captured Southeast China, accounting for 93.6% (1047/1119) of the total fleas. A total of 308 fleas were from Zhangzhou city, 191 fleas were from Quanzhou city, 127 fleas were from Sanming city, 60 fleas were from Longyan city, 127 fleas were from Ningde city, 34 fleas were from Fuzhou city and 272 fleas were from Putian city.

**Table 1:** Flea collection from seven cities in Southeast China

Flea species	Location							Total
	Zhangzhou	Quanzhou	Sanming	Longyan	Ningde	Fuzhou	Putian	
*Xenopsylla cheopis*	306	190	92	57	114	24	264	1047
*Monopsyllus anisus*	–	–	34	1	3	–	–	38
*Leptopsylla segnis*	2	–	1	2	2	10	7	24
*Nosopsyllus nicanus*	–	1	–	–	–	–	–	1
*Ctenocephalides felis*	–	–	–	–	5	–	1	6
*Stivalius klossi bispiniformis*	–	–	–	–	2	–	–	2
*Neopsylla dispar fukienensis*	–	–	–	–	1	–	–	1
Total	308	191	127	60	127	34	272	1119

Values in table are the number of fleas of each species collected per location

### Detection and quantification of *Bartonella* spp. DNA

*Bartonella-ssrA* DNA was detected in 26.2% (293/1119, 95% confidence interval [CI] 23.6–28.8%) of the tested fleas from Southeast China (Table 2). Among the fleas found, 27.1% (284/1047) of the *X. cheopis*, 13.2% (5/38) of the *M. anisus*, 8.3% (2/24) of the *L. segnis* and 20.0% (2/10) of the ‘other’ fleas (*N. nicanus*, *C. felis*, *S. klossi bispiniformis* and *N. fukienensis*) were positive for *Bartonella*, with *X. cheopis* having the highest prevalence of infection and *L. segnis* the lowest. There was a significant difference in the prevalence of *Bartonella* among the different flea species (*χ*^2^ = 9.48, df = 3, *P* = 0.024). The infection rate of female fleas (28.9%, 217/750) was greater than that of male fleas (20.6%, 76/369), and there was a significant difference in the prevalence of infection between the sex (*χ*^2^ = 8.89, df = 1, *P* = 0.003).

**Table 2:** Molecular detection of *Bartonella* species in fleas from rats collected in Southeast China

Effect	Location							Total (%)	*χ*^2^ value	*P* value
	Zhangzhou	Quanzhou	Sanming	Longyan	Ningde	Fuzhou	Putian			
Species									9.48	0.024
X. cheopis	78/306	89/190	22/92	20/57	37/114	3/24	35/264	284/1047 (27.1%)		
M. anisus	–	–	5/34	0/1	0/3	–	–	5/38 (13.2%)		
L. segnis	0/2	–	0/1	0/2	½	0/10	1/7	2/24 (8.3%)		
‘Other’	–	0/1	–	–	2/8	–	0/1	2/10 (20%)		
Sex									8.89	0.003
Female	53/201	60/116	19/78	16/45	36/94	3/28	30/188	217/750		
Male	25/107	29/75	8/49	4/15	4/33	0/6	6/84	76/369		
Host species									18.948	0.008
Rattus norvebicus	37/148	80/152	13/58	12/33	30/97	0/10	20/146	192/644		
Rattus flavipectus	32/131	1/14	13/57	6/22	10/26	2/14	14/111	78/375		
Rattus losea	3/7	–	0/1	–	0/2	0/2	0/3	3/15		
Niviventer coninga	–	–	0/7	–	–	–	–	0/7		
Bandicota indica	–	–	–	–	0/2	–	–	0/2		
Mus musculus	0/1	–	–	½	–	0/2	0/1	1/6		
Niviventer fulvescens	–	0/1	–	–	–	0/3		0/4		
Suncus murinus	6/14	8/24	1/4	–	–	½	2/11	18/62		
Missing data	0/1	–	–	1/3	–	–	–	1/4		
Habitats									44.62	< 0.001
Residential	77/305	–	27/127	19/57	40/125	3/29	36/272	202/915		
Wildernesses/farmland	½	89/191	–	–	0/2	0/5	–	90/200		
Missing data	0/1	–	–	1/3	–	–	–	1/4		
Total	78/308 (25.3%)	89/191 (46.6%)	27/127 (21.3%)	20/60 (33.3%)	40/127 (31.5%)	3/34 (8.8%)	36/272 (13.2%)	293/1119 (26.2%)		

Values in table are presented as the number of Bartonella-infected fleas/total fleas collected per flea species, region, gender, host species and habitat

In this study, seven rodent species, namely* Rattus norvegicus*,* Rattus flavipectus*,* Rattus losea*,* Niviventer coninga*,* Bandicota indica*,* Mus musculus*, and* Niviventer fulvescens*, and one mammal species,* Suncus murinus*, were captured. When fleas from* N. coninga*,* B. indica* and* N. fulvescens* were not taken into account, the prevalence of fleas ranged from 16.7% to 29.8% (note: host species was not recorded in four fleas; Table 2). There was a significant difference in the prevalence among different hosts (*χ*^*2*^ = 18.948, df = 7, *P* = 0.008). Two or more fleas were captured from 245 hosts, with 14.5% (33/245) of these infected with* Bartonella*; 30.6% (75/245) were infected by only one flea and 55.9% (137/245) were not infected.

There was a significant difference in the prevalence of *Bartonella* in the different regions (*χ*^2^ = 75.23, df = 6, *P* < 0.001), with the highest incidence (33.3%, 20/60) occurring in Longyan City and the lowest prevalence (8.8%, 3/34) occurring in Fuzhou City (Table 2). In terms of geographical location of the seven cities investigated, Ningde City, Fuzhou City, Putian City, Zhangzhou City and Quanzhou City are located in the coastal area, and Sanming City and Longyan City are located in the inland area. The prevalence of* Bartonella* in the coastal cities was 26.4% (246/932) and that in the inland cities was 25.1% (47/187); the difference in prevalence among these two different geographic locations was not statistically significant (*χ*^2^ = 0.13, df = 1, *P* > 0.05). With the exception of four fleas from unrecorded habitats, 22.1% (202/915) of the fleas collected in wildernesses/farmlands were infected with *Bartonella*, and 45% of fleas (90/200) collected in residential areas were infected (Table 2). Fleas from residential areas had a significantly greater prevalence of *Bartonella* infection than did those collected in fields/farmland (*χ*^2^ = 44.62, df = 1, *P* < 0.001).

**Fig. 1:**
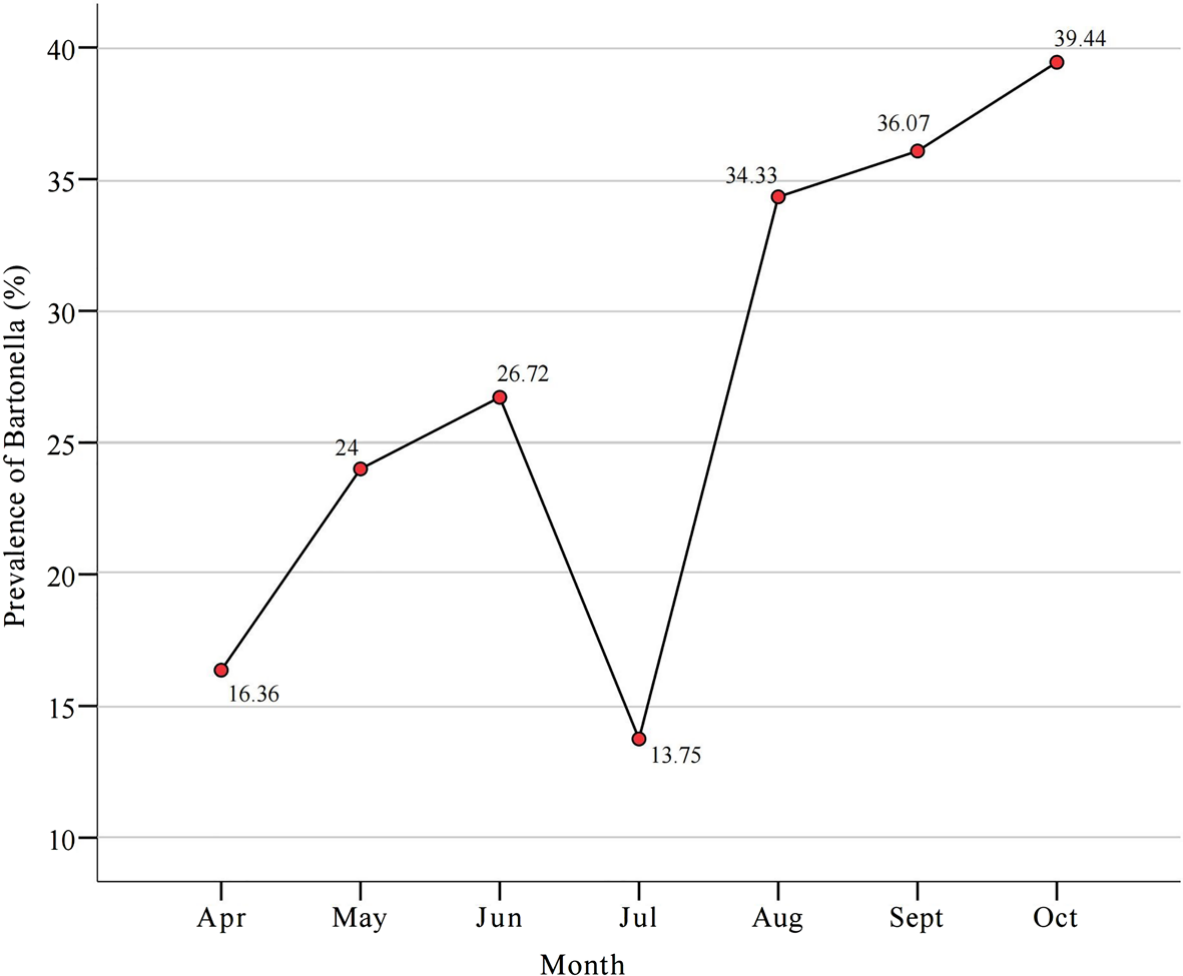
Monthly prevalence of *Bartonella* in fleas in southeast China. Filled circles represent the prevalence of *Bartonella*

The prevalence of *Bartonella* in flea samples showed seasonal variation (Fig. 1), increasing from 16.4% in April to 26.7% in June, then decreasing to 16.8% in July, followed by an increase to a peak infection of 39.4% from August to October. The difference in *Bartonella* prevalence was significantly different between the different months (*χ*^2^ = 32.08, df = 6, *P* < 0.001).

**Fig. 2:**
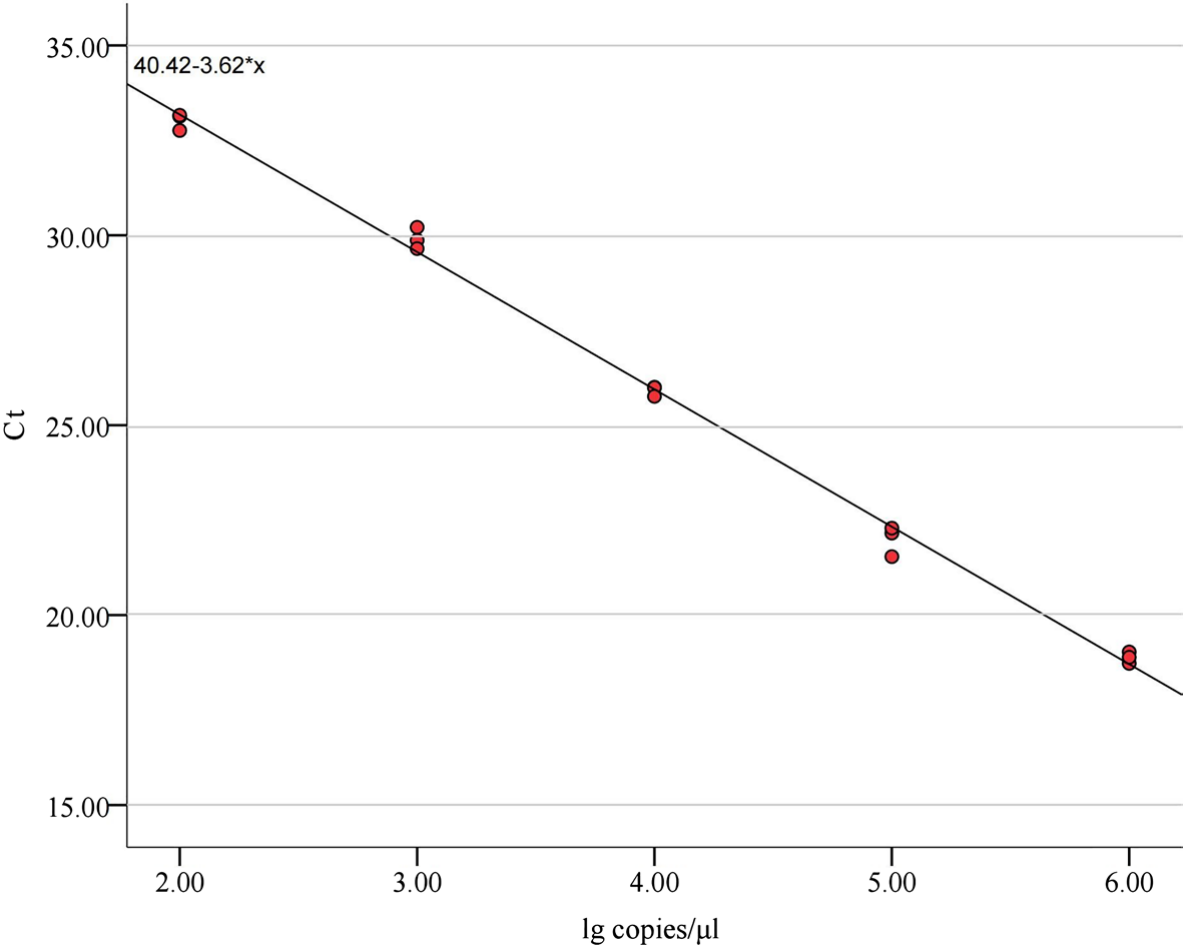
Standard curve based on *Bartonella henselae ssrA* gene fragment plasmid DNA. Tenfold serial dilutions of the plasmid vector DNA were performed (2log_10_-6log_10_ copies/μl), and real-time PCR analyses were repeated three times for each dilution concentration. The slope and intercept of the regression curve are shown. Ct Cycle threshold; lg, log

**Fig. 3:**
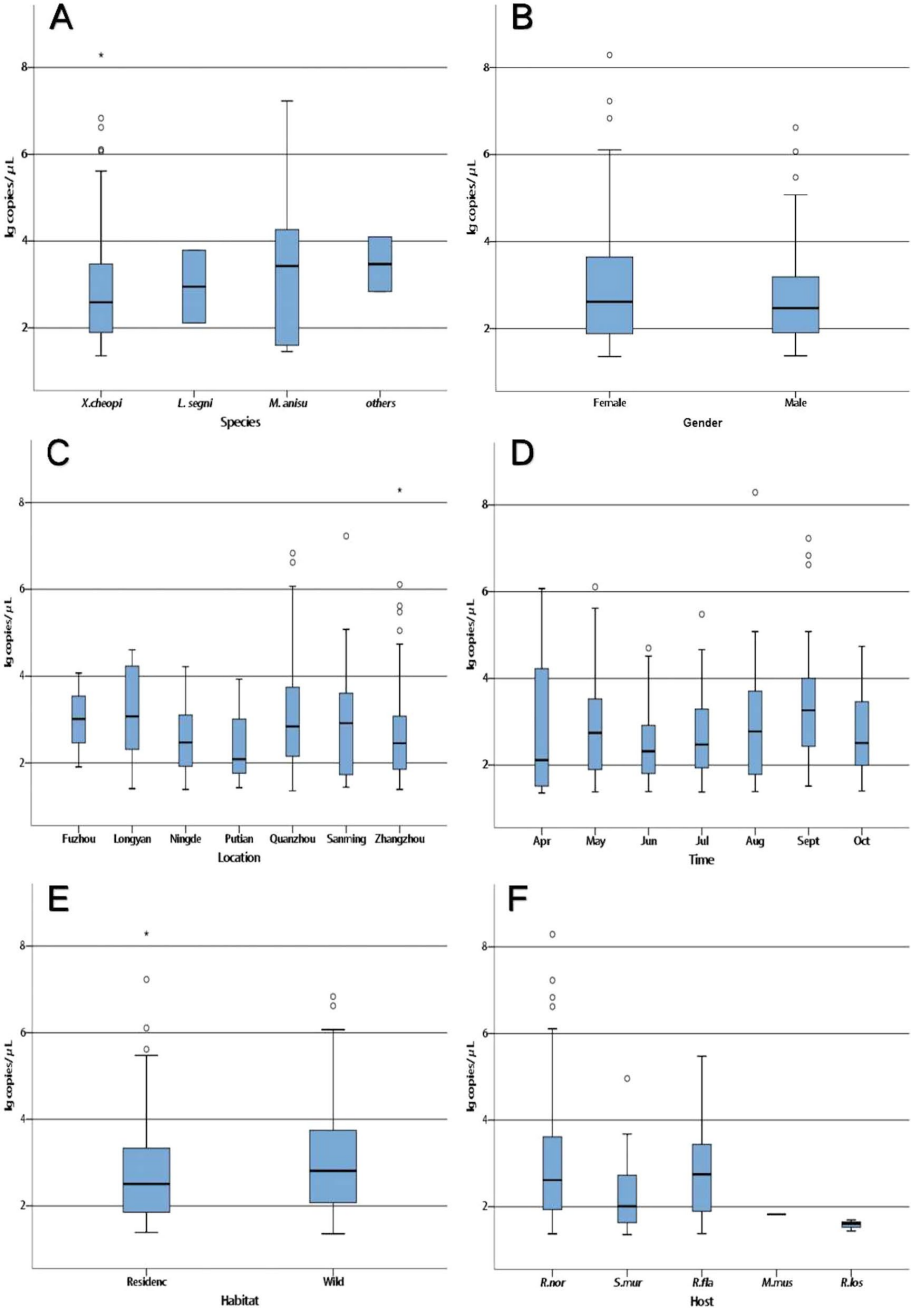
Boxplot of *Bartonella* loads in positive samples from fleas of different species (**a**), sex (**b**), locations (**c**), time points (**d**), habitats (**e**) and hosts (**f**). Boxes represent IQRs, and vertical lines represent the distribution of maximum and minimum values. The values on the* y*-axis are expressed as log DNA copies/μl

A standard curve was established using plasmid DNA from the *B. henselae ssrA* gene fragment with* r*^2^ = 0.996, a slope of − 3.62, and a* y*-intercept of 40.42 (Fig. 2). The *Bartonella* loads of the positive fleas ranged from 1.35 to 8.29 log_10_ copies/μl (mean ± standard deviation [SD] 2.78 ± 1.14). Flea *Bartonella* loads were statistically significantly different among the different regions (F = 2.178, *P* = 0.045), with the highest flea bacterial loads occurring in Longyan city (mean ± SD, 3.19 ± 1.13) and the lowest occurring in Putian city (mean ± SD, 2.34 ± 1.14) (Fig. 3c). The fecal bacterial load in fleas caught in wildernesses (mean ± SD, 2.99 ± 1.20) was significantly higher than that in fleas caught in residential areas (mean ± SD, 2.70 ± 1.11) (t = − 2.010, *P* = 0.045) (Fig. 3e). Changes in flea bacterial loads over time showed a trend similar to that of prevalence and were significantly different (F = 3.148, *P* = 0.005) (Fig. 3d). Differences in flea *Bartonella* loads among flea species (F = 1.108, *P* = 0.346) (Fig. 3a), sex (t = 0.553, *P* = 0.581) (Fig. 3b) and host species (F = 1.977, *P* = 0.098) (Fig. 3f) were not statistically significant.

### Sequence comparison and phylogenetic analysis

**Fig. 4:**
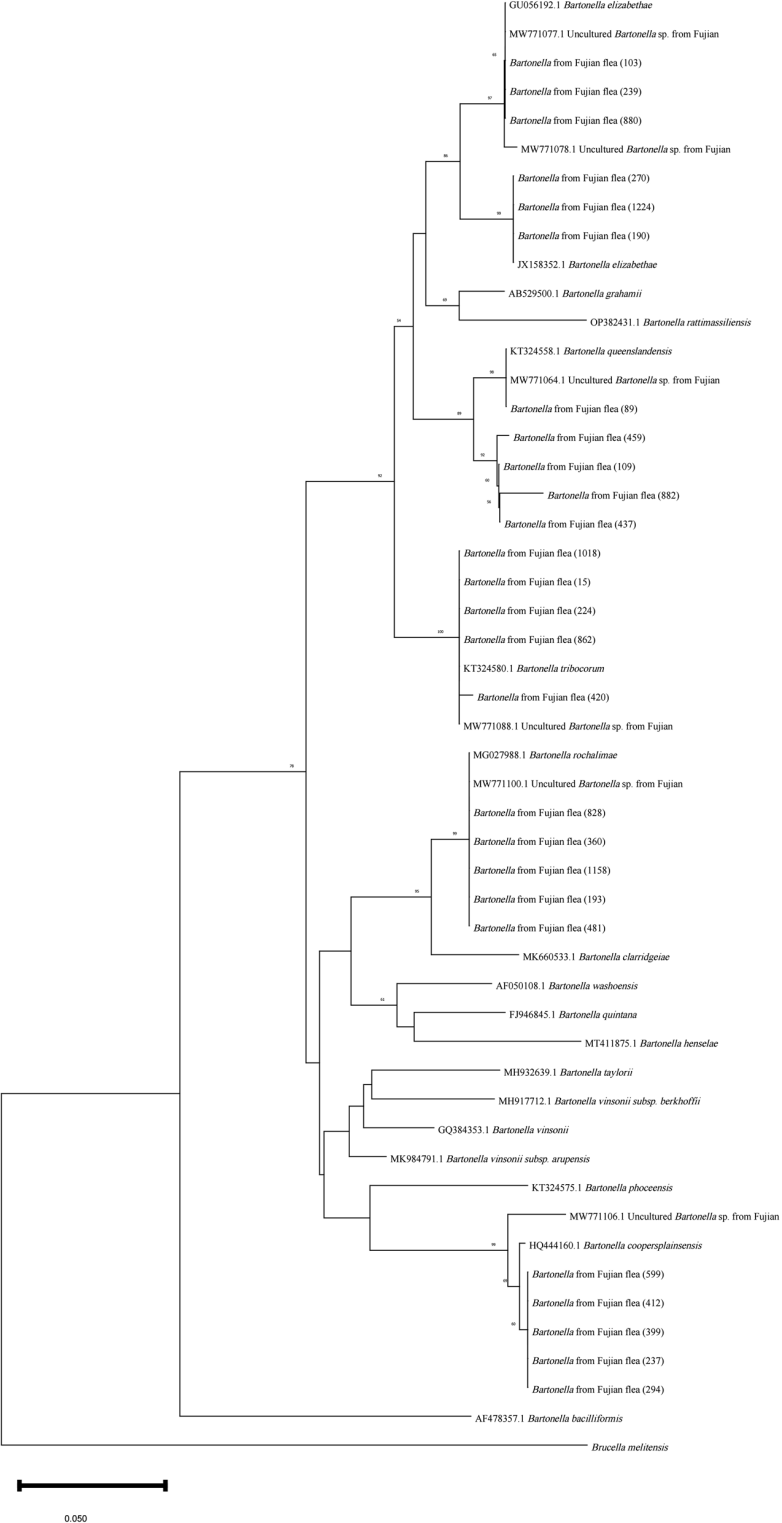
Phylogenetic tree based on the *gltA* gene of *Bartonella*. The phylogenetic tree was constructed using the neighbor-joining method based on the maximum composite likelihood model, and bootstrap values were calculated with 1000 replicates

In total, 114 *gltA* sequences were analyzed via BLAST, and the phylogenetic analysis included sequences of 18 *Bartonella* genotypes, six *Bartonella* strains previously isolated from rodents in Southeast China and 26 representative flea samples from the present study. *Brucella* was also included as an outgroup (Fig. 4). The phylogenetic tree showed that the *Bartonella*-positive samples could be divided into five different branches. A total of 35.1% (40/114) of the *gltA* sequences belonged to *B. tribocorum*, which is the dominant genotype in Southeast China and is in the same branch as KT324580 in Thailand and MW771088 in Fujian, with 100% similarity. Seven sequences were detected as *B. queenslandensis*, with 95.5%-100% similarity to KT324558 from Thailand and MW771064 from Fujian. Twenty-three sequences of *B. elizabethae* were 99.1–100% homologous to JX158352 and GU056192 from Thailand and Taiwan, as well as to MW771077 and MW771078 from Fujian. Twenty-seven *B. rochalimae* sequences showed 100% similarity with those of MG027988 from the USA and MW771100 from Fujian. Nine *B. coopersplainsensis* sequences showed 94.3–98.5% similarity with HQ444160 from Australia and MW771106 from Fujian. Although the previously investigated rodents were not the hosts of the present flea samples, their *Bartonella* spp. were analyzed against the present samples, and the similarity reached 96.1–100%. Interestingly, of two or more fleas from the same host, four pairs were infected with the same *Bartonella* species: *B. tribocorum*, *B. rochalimae* and *B. elizabethae*.

The differences in flea *Bartonella* loads among the different regions were significantly different (*P* < 0.001). *Bartonella coopersplainsensis*-infected fleas were found to have higher bacterial loads (mean ± SD, 3.92 ± 0.57 log_10_ copies/μl) than other species. Among all species, *B. elizabethae* had the lowest load (mean ± SD, 2.13 ± 0.566 log_10_ copies/μl).

## Discussion

Fleas are recognized as key players in the transmission of *Bartonella*, as they are able to carry a high diversity of *Bartonella* species and transmit them efficiently among rodents [26]. This efficient transmission of *Bartonella* is regarded as an important factor in maintaining its high prevalence in the natural environment. In China, there are relatively few investigations on ectoparasite infections caused by *Bartonella*. Li DM [27, 28], who detected *Bartonella* from the bacteria *Chlamydophila felis* and *Leptopsylla segnis*, isolated *Bartonella* strains from fleas and ticks. *Bartonella* infection in fleas has also been found in Qinghai Province, the Qinghai-Tibet Plateau and the China-Kazakhstan Border [29–31]. The present study emphasized the prevalent distribution of *Bartonella* in fleas and the related genotypes in Southeast China, with the data showing that there was a high prevalence of *Bartonella* in fleas in Southeast China and that multiple *Bartonella* genotypes could be identified.

The reported prevalence of flea *Bartonella* DNA detection in various countries varies, ranging from 2.2% to 40% in Egypt, the USA, France, Chile and Japan [9, 14, 32–34]. The overall *Bartonella* infection rate in fleas in the present study was 26.2% (293/1119) according to the qPCR analyses, which is higher than that reported our previous study of *Bartonella* infection in rodents in Southeast China (14.6–14.9%) [19, 35]. One factor for fleas possessing such a high infection rate may be their frequent feeding and ability to move from one host to another [36]. Our results also showed that *B. tribocorum* was the predominant genotype of *Bartonella* fleas in Southeast China; this species can cause causing acute fever and bacteremia in humans. Therefore, it is necessary to evaluate the epidemiological characteristics of *Bartonella* in fleas.

The transmission and acquisition of *Bartonella* are mediated by the host specificity of fleas, flea exchange between rodents and flea abundance [37]. According to our survey, *X. cheopis* was the dominant flea species in Southeast China and also the most prevalent flea. Our observations are similar to the results of a survey of rodents in the USA [38], which showed that the highest prevalence usually occurred among the most common species in rodent communities. These results indicate that the increase in the prevalence of *Bartonella* in fleas may also be related to the dominance of flea species in the population. Moreover, the density of the hosts may also increase flea transmission and infection among the hosts, as we mainly conducted surveys in villages and surrounding farmland where captured rodents were dominated by domestic rats, such as *R. norvebicus*, *R. flavipectus* and *S. murinus*, which were also accompanied by a high prevalence of parasitic flea infestations on their body surfaces (20.8–29.8%). Additionally, we found that residential areas have higher infection rates than wildernesses/farmlands, which undoubtedly increases the likelihood of flea contact with humans and disease transmission. In addition, animal sex has not been identified as a risk factor for *Bartonella* infection in rodents from Taiwan and France [39, 40]. However, in our study, we found that the incidence of *Bartonella* infection was significantly greater in females than in males, and this difference may be related to the parasitism and blood-sucking habits of fleas, with females sucking a greater amount of blood more frequently and for a longer period than males.

Several previous studies have shown that the prevalence of *Bartonella* in rodents and their ectoparasitic fleas is influenced by seasonality, peaking from the summer to fall [41–43]. In the present study, we found that the prevalence of *Bartonella* in fleas was markedly seasonal, with a clear upward trend in the prevalence of this genus from July to October. Late summer and early fall are not only periods of prevalence of *Bartonella* transmission but also periods of peak vector activity [44], making this period a risky time for *Bartonella* transmission to other species, including humans.

Phylogenetic analysis of the *Bartonella gltA* gene revealed five *Bartonella* genotypes, namely *B. tribocorum*, *B. queenslandensis*, *B. elizabethae*, *B. rochalimae* and *B. coopersplainsensis*, indicating the high diversity of *Bartonella* in the fleas of Southeast China. *Bartonella tribocorum*, *B. elizabethae* and *B. rochalimae* were the major genotypes identified in this survey, and all of them are pathogenic to humans, causing endocarditis, myocarditis, fever and neurological diseases. The high diversity of *Bartonella* genotypes may be a result of frequent host changes in fleas and their high efficiency in transmitting *Bartonella*. We compared the sequences of *Bartonella* species previously isolated from rodents in Southeast China with those isolated in the present study; the homology was 96.2%-100%, indicating the high adaptation of *Bartonella* species to rodents and fleas. In addition, Bowen et al. [11] reported that 75% (21/28) of bank voles housed with wild-caught fleas for 4 weeks developed *Bartonella* infections, and the present study also revealed multiple groups of fleas from the same host infected with the same *Bartonella* genotype at the same time, suggesting that fleas may play a potential role as vectors for the transmission of *Bartonella* among rodents. However, it is worth noting that the PCR detection of *Bartonella* spp. in fleas does not necessarily mean that they actively infest the host. Consequently, the mechanism of *Bartonella* spp. transmission between fleas and rodents still needs to be investigated more thoroughly.

## Conclusions

The present study describes the prevalence and genetic characteristics of *Bartonella* species in fleas in southeast China. The results showed that there was a high prevalence and diversity of *Bartonella* in fleas. We identified five *Bartonella* genotypes in fleas, of which the zoonotic *B. tribocorum, B. elizabethae*, and *B. rochalimae* will pose a threat to human health in southeast China. However, the vector capacity of fleas was not determined in this study. In future studies, the host-vector relationship of *Bartonella* can be further investigated via animal experiments.

### Supplementary Information

**Additional file 1: Table S1.** Rodent species and their ectoparasitic flea species.

## Data Availability

All data generated or analysed during this study are included in this published article [and its supplementary information files].

## Abbreviation

qPCR: Quantitative real-time PCR
